# A Photoprotein in Mouse Embryonic Stem Cells Measures Ca^2+^ Mobilization in Cells and in Animals

**DOI:** 10.1371/journal.pone.0008882

**Published:** 2010-01-27

**Authors:** Silvia Cainarca, Simone Fenu, Cinzia Ferri, Cinzia Nucci, Patrizia Arioli, Andrea Menegon, Lorenzo Piemonti, Stefan Lohmer, Lawrence Wrabetz, Sabrina Corazza

**Affiliations:** 1 Axxam SpA, Milan, Italy; 2 Division of Genetics and Cell Biology, San Raffaele Scientific Institute, DIBIT, Milan, Italy; 3 ALEMBIC (Advanced Light and Electron Microscopy Bio-Imaging Centre), San Raffaele Scientific Institute, Milan, Italy; 4 San Raffaele Diabetes Research Institute (HSR-DRI), San Raffaele Scientific Institute, Milan, Italy; Instituto Butantan, Brazil

## Abstract

Exogenous expression of pharmacological targets in transformed cell lines has been the traditional platform for high throughput screening of small molecules. However, exogenous expression in these cells is limited by aberrant dosage, or its toxicity, the potential lack of interaction partners, and alterations to physiology due to transformation itself. Instead, primary cells or cells differentiated from precursors are more physiological, but less amenable to exogenous expression of reporter systems. To overcome this challenge, we stably expressed c-Photina, a Ca^2+^-sensitive photoprotein, driven by a ubiquitous promoter in a mouse embryonic stem (mES) cell line. The same embryonic stem cell line was also used to generate a transgenic mouse that expresses c-Photina in most tissues. We show here that these cells and mice provide an efficient source of primary cells, cells differentiated from mES cells, including cardiomyocytes, neurons, astrocytes, macrophages, endothelial cells, pancreatic islet cells, stably and robustly expressing c-Photina, and may be exploited for miniaturized high throughput screening. Moreover, we provide evidence that the transgenic mice may be suitable for *ex-vivo* bioimaging studies in both cells and tissues.

## Introduction

Movements of Ca^2+^ ions are fundamental for signal transduction in cells. Because the intracellular level of Ca^2+^ is highly regulated and compartmentalized, transient alterations in Ca^2+^ concentration are excellent signals, and are downstream targets of G-protein coupled receptors (GPCRs), ion channels and transporters, all important examples of therapeutic targets [1]. One effective tool to measure Ca^2+^ mobilization sensitively, and non-invasively, are photoproteins, which release photons upon binding to Ca^2+^ (in presence of a cofactor). These photoproteins are widely used in cell-based assays for high throughput screening (HTS) as reporter genes to monitor Ca^2+^ movements associated with signals [2]–[4]. The sensitive detection, virtually undetectable background and high signal to noise ratio favour photoproteins over Ca^2+^-sensitive fluorescent dyes and permit small assay-volumes [5], [6]. The cofactor, coelenterazine, is added to mammalian cells expressing the photoprotein and photon emission is detected as an indicator of intracellular Ca^2+^ concentration [7].

Usually, cell-based assays exploit transformed cell lines, which express both a photoprotein and a target receptor. These cell lines have been selected for limited expression of other receptors, for their easiness to be cultured, and to be expanded. However, exogenous expression of targets and the transformed environment can create artefacts of gene dosage, toxicity, or stoichiometry of the receptor target itself, when it requires assembly of multiple subunits. An alternative to transformed cell lines are primary cells, isolated from mammals. They have a more physiological environment and may express targets endogenously, but they are frequently complicated to purify and culture in sufficient numbers, and furthermore, are sometimes very difficult to transfect with (reporter) genes.

The embryonic stem cells are a possible alternative. By maintaining the self-renewal property of the undifferentiated state, they can be cultured and expanded *in vitro* for long periods, and they are quite easily transfected [8]. Moreover, embryonic stem cells can differentiate into virtually any cell type, resembling primary cells [9], [10]. Accordingly, they offer a natural environment for the receptor targets, and they can form stoichiometrically appropriate complex targets (like multi-subunit ion channels), that are regulated natively [11].

Therefore, we generated clones of mouse embryonic stem cells expressing a photoprotein as a Ca^2+^ reporter system under the control of a ubiquitous promoter. We show that multiple types of cells differentiated from one of these clones report Ca^2+^ signals in response to physiological stimuli. Furthermore, we exploited these undifferentiated photoprotein mES cells to produce a transgenic mouse, which may be useful for *ex vivo* imaging studies and as a source of differentiated primary cells expressing a Ca^2+^ reporter gene.

## Results

### 1. c-Photina Photoprotein

To identify a photoprotein which combines sensitive detection of intracellular mobilized Ca^2+^ and stable expression, we performed random mutagenesis of *Clytin*, a natural photoprotein isolated from *Clytia gregaria* jellyfish (syn. with *Phialidin*) [12], [13]. One of those modified photoproteins displayed the desired characteristics and was called c-Photina®. To improve the transduction efficiency in mammalian cells, the *c-Photina* gene was optimized for mammalian codon usage (as described for *Photina®* in [6], and then fused to a mitochondrial tag (human *Cytochrome C Oxidase*, subunit VIII) [14], in an expression vector that contained no antibiotic resistance gene.

**Figure 1:**
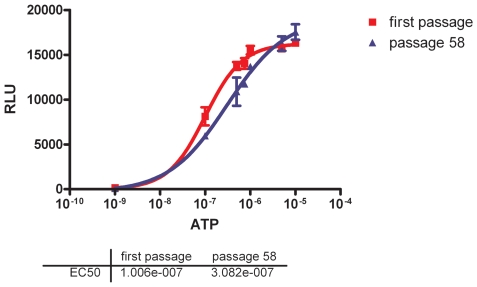
ATP dose response curves in CHO c-Photina cells at different passages. The experiments were performed using 500 cells/well in 384 MTP 24 hours after seeding. *Lumibox luminometer conditions: low sensitivity, reading time 30 seconds*.

To investigate the function and stability of *mito c-Photina* expression, we transfected CHO-K1 cells, in order to create a stable clone [6]. The cells were kept in culture for more then 7 months and 58 passages, in absence of antibiotic selective pressure, and we confirmed function regularly by stimulating with agonists for endogenous GqPCRs (Gαq Protein Coupled Receptors), whose activation induce a Ca^2+^ release from internal stores through the Gαq/phospholipase C pathway. The kinetics of the bioluminescent response indicated high affinity for Ca^2+^, and stable expression over time, as specified by the consistent EC_50_ for ATP (Figure 1). This was an important feature since several mammalian cells transfected with other natural or recombinant photoproteins, tended to loose function over time in the absence of selective pressure (unpublished). The stability of mito c-Photina function made this mutant the first choice for transfection into mES cells.

### 2. Generation of a mES Cell Line Expressing a Photoprotein

**Figure 2:**
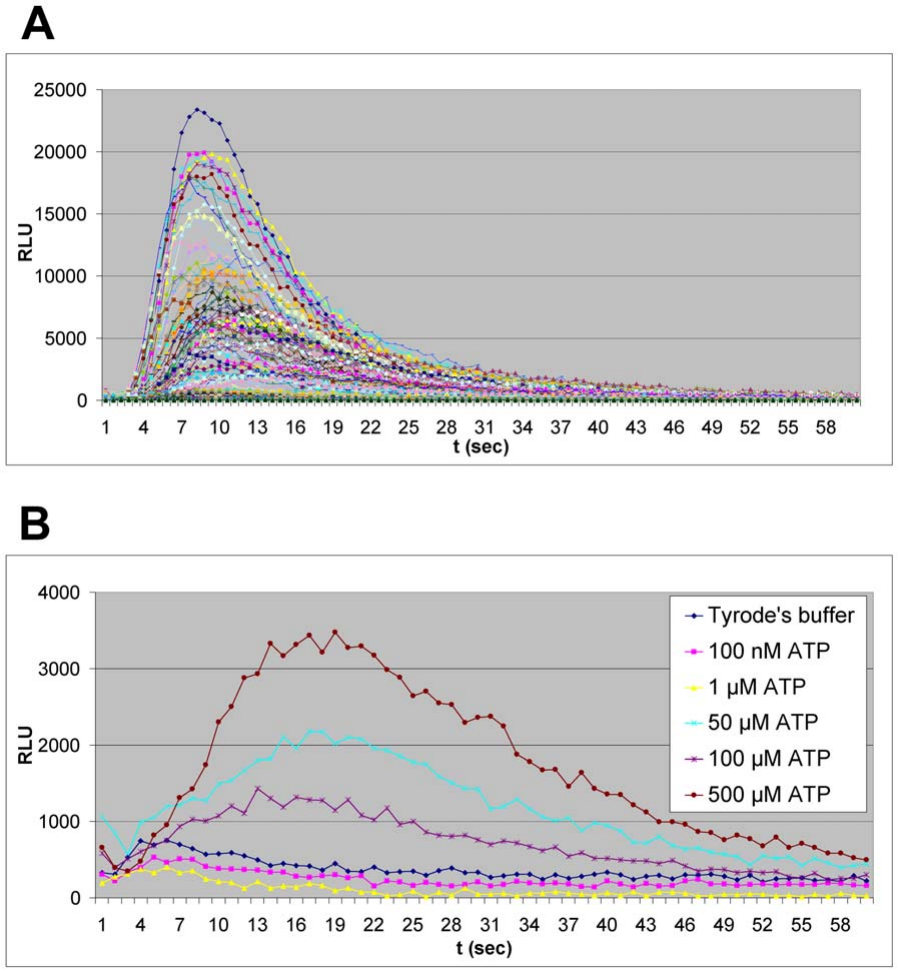
c-Photina mES clone selection. **A.** Clone pool analysis of the 114 neomycin resistant clones. Histamine response kinetics (100 µM) were measured at Lumibox luminometer and recorded as RLU (Relative Luminescence Units) values. The experiments were performed in 96 MTP 24 h after cell seeding. *Lumibox luminometer conditions: high sensitivity, for 60 seconds.* **B.** mES/mito c-Photina/29 clone histamine dose response. The histamine response (100 nM–500 µM) was measured in 96 MTP 24 h after seeding 20,000 cells/well. *Lumibox luminometer conditions: high sensitivity, reading time 60 seconds*.

We electroporated mouse ES cells with the *mito c-Photina* gene. After neomycin selection, 114 drug-resistant colonies were picked, and expanded on mouse embryonic fibroblasts (MEFs), and screened for the ability to emit light after functional stimulation with histamine, which is known to activate the endogenously expressed GqPCR histamine-1 receptor in mES cells [15] and consequently to raise transiently the cytoplasmic Ca^2+^ concentration. A typical GqPCR-mediated response curve [6] was obtained after injection of 100 µM histamine from almost all the clones (Figure 2A).

To verify that the response amplitude correlated with amount of photoprotein, cells, after primary measurement, were lysed to expose all coelenterazine-c-Photina complexes to Ca^2+^. This response was much higher than that of all clones (data not shown) and was not always correlated to the differences in amplitude observed after GqPCR stimulation, suggesting that total coelenterazine-c-Photina reacting complex was not limiting.

The final mES mito c-Photina clone was selected from 12 high responders on the basis of different parameters: primarily the ability to respond to histamine in a dose-responsive manner normalized for cell number, secondly the total photoprotein content after cell lysis, but also the number of copies of the transgene in the host genome, karyotype, cell morphology and growth rate. The number of copies in the genome was analyzed by Southern blot and quantitative PCR analysis. Clone 29 was selected on the basis of the parameters described before (see its histamine dose-responsiveness in Figure 2B) and because it has the transgene inserted into the genome in a single copy. Further confirmation of this was the impossibility to detect signals by FISH analysis (data not shown).

### 3. mES/mito c-Photina/29 Clone Is Pluripotent

**Figure 3:**
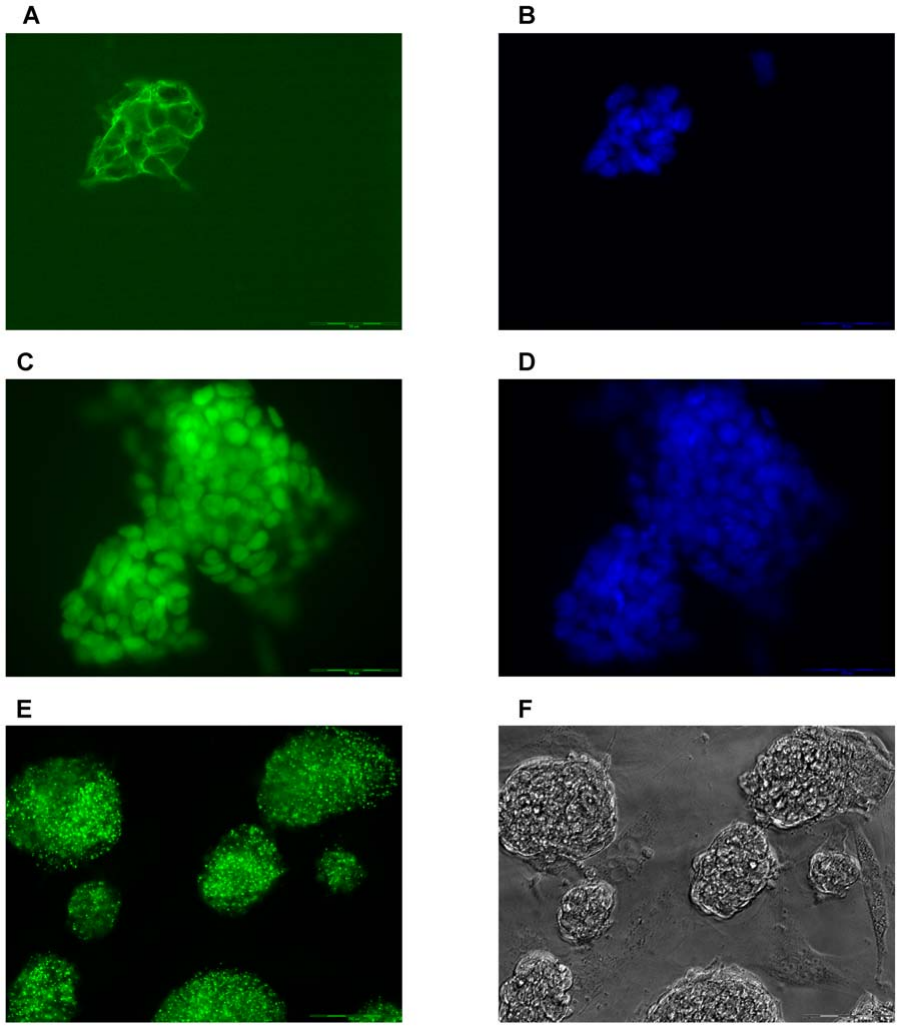
Immunofluorescence analysis on undifferentiated mES/mito c-Photina/29 clone. The assay was performed using the following antibodies: **A. B.** Anti-SSEA-1 primary antibody (**A.**), counterstained with Hoechst 33342 dye (**B.**). Scale bar -50 µm. **C.-D.** Anti-oct 3/4 primary antibody (**C.**), counterstained with Hoechst 33342 dye (**D.**). Scale bar -50 µm. **E.-F.** Alkaline phosphatase activity measured with the ELF® Phosphatase fluorescent staining kit (**E.**) and its corresponding contrast phase image (**F.**). Scale bar -200 µm.

Indirect immunofluorescence assays were performed on the mES mito c-Photina clone in order to evaluate the presence of specific markers of the undifferentiated pluripotent mouse embryonic stem cells, such as the stage specific embryonic antigen-1 (SSEA-1) [16] (Figure 3A–B) and the transcription factor oct 3/4 [17] (Figure 3C–D). As shown in Figure 3 both SSEA-1 and oct 3/4 are present selectively in stem cells and not in the surrounding feeder cells. Additionally, we could detect also alkaline phosphatase activity (Figure 3E–F), another characteristic of undifferentiated stem cells [18].

Since the “*bona fide*” demonstration of stemness is the germline transmission test, clone 29 was injected into blastocysts of pregnant host female mice. Two chimeric mice, with a high degree of chimerism (almost 100%) and male phenotypes, were obtained. When these 2 mice reached sexual maturity, they were crossed with C57BL/6 female mice and gave rise to more than 95% agouti progeny (Table 1), indicating robust germline transmission.

**Table 1:** Germline transmission results for clone 29 c-Photina mouse embryonic stem cells.

	CHIMERA Nr.1		CHIMERA Nr.2	
Litter	nr agouti mice	nr c-Photina positives	nr agouti mice	nr c-Photina positives
**I**	10	4	4	2
**II**	5	1	7	5
**III**	12	6	9	7
**IV**	10	4		
**V**	6	3		

List of agouti and c-Photina mice born from the 2 chimeric males crossed with C57BL/6 female mice, indicating the germline transmission.

### 4. In Vitro Differentiation Assays Performed with mES/mito c-Photina/29 Clone

To show that the introduction of the transgene did not influence the “*in vitro*” differentiation capabilities of the mito c-Photina/29 clone, we cultured the cells under conditions to favour either cardiomyocyte or neuronal fates, employing well described protocols including suspension protocols for embryoid body (EB) formation, and adhesion protocols [19], [20] all optimized for miniaturized formats.

#### 4.1. Cardiomyocytes

Cardiomyocytes are one of the most important cell types for drug discovery projects and the hallmark of cardiomyocytes is the Ca2+ dependent contractility and its characteristic Ca2+ channel driven depolarisation curve. In addition, the heart rate is controlled by GqPCR-dependent Ca2+ release, for example mediated by the adrenergic receptors.

**Figure 4:**
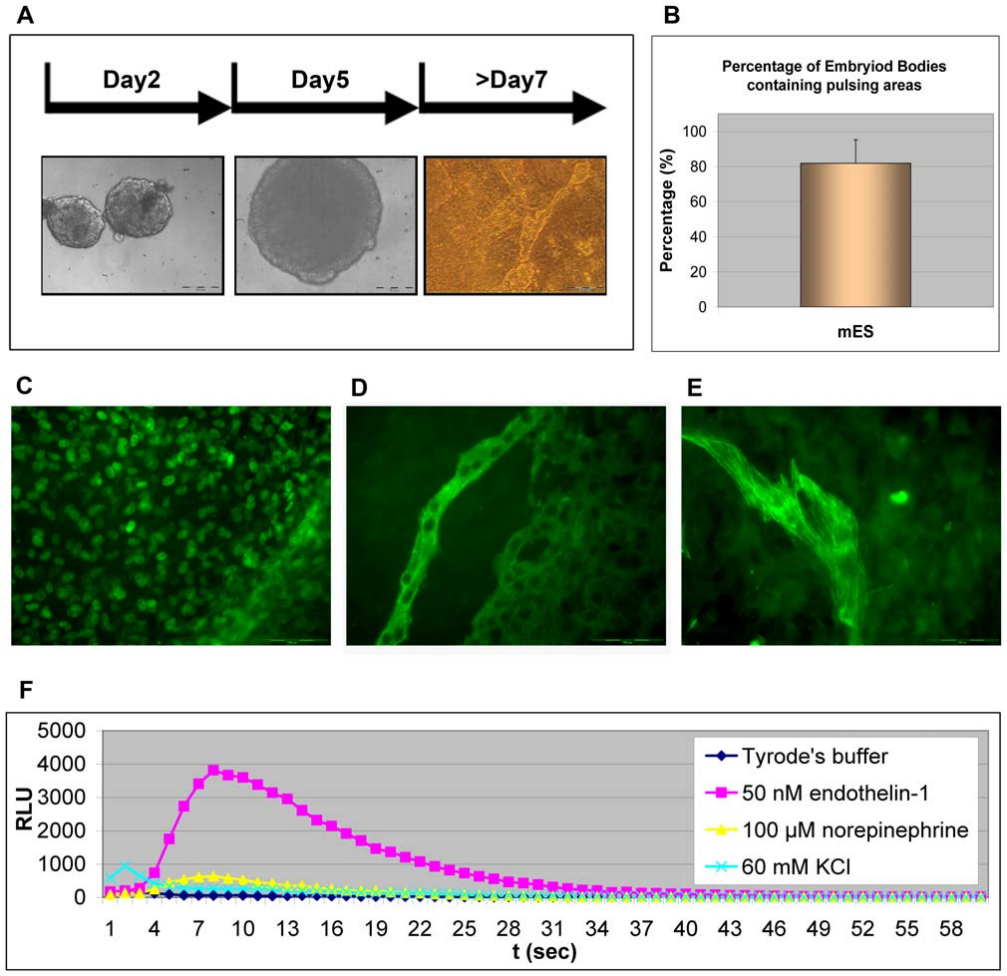
A–B. *In vitro* cardiomyocyte differentiation assay on mES/mito c-Photina/29 clone. **A.** Embryoid bodies in suspension at differentiation day 2 (contrast phase image). Embryoid body in suspension at differentiation day 5 before the plating on gelatin-coated dishes (contrast phase image). Embryoid body in adhesion at differentiation day 7 on gelatin-coated dishes (contrast phase image). Scale bar -200 µm. **B.** Percentage of embryoid bodies containing pulsing areas obtained after morphological analysis (see also Videos S1 and S2). **C.-E.** Immunofluorescence analysis on *in vitro* differentiated cardiomyocytes from mES/mito c-Photina/29 clone. The immunofluorescence assay was performed using the following antibodies: **C.** Anti-GATA-4 primary antibody. Scale bar -100 µm. **D.-E.** Anti-alpha myosin heavy chain primary antibody. Scale bar −100 µm (**D.**) and −50 µm (**E.**). **F.** CCD camera-based functional test on *in vitro* differentiated cardiomyocytes from mES/mito c-Photina/29 clone. Tyrode's buffer, endothelin-1 (50 nM) and norepinephrine (100 µM), and KCl (60 mM) responses were measured in 384 MTP 48 h after 5,000 cells/well seeding. *Lumibox luminometer conditions: high sensitivity, reading time 60 seconds*.

EBs were formed in hanging drops for two days and then in suspension for another three days. The fifth day, EBs were plated on gelatin-coated tissue culture dishes. Within one day, we observed spontaneously pulsating cardiomyocytes. The percentage of EBs containing pulsating areas was about 80% (Figure 4A–B, and Video S1 and Video S2). The protocol was adapted to miniaturized formats, putting exactly a single EB per well of 96 or 384 micro titre plates (MTP). The cardiomyocyte development occurred directly in the micro titre plate format, maintaining the same proportion of pulsating areas.

To verify the presence of mature cardiomyocytes we stained for specific cardiomyocyte markers, such as the transcription factor, GATA-4 (Figure 4C), and for the cytoskeleton protein, myosin heavy chain (MHC) (Figure 4D-E). As seen in Figures 4C, D, and E all the markers were present and showed appropriate localization demonstrating proper cardiomyocyte development.

Next, functional tests were performed by disaggregating EBs and seeding 5,000 cells/well in a 384 MTP under the same differentiation conditions. 48 hours after seeding, the cells were stimulated with standard Tyrode's buffer as control, 50 nM endothelin-1, and 100 µM norephinephrine, which are agonists for the endogenously expressed GqPCR endothelin receptors and for the α1-adrenergic receptor, respectively. The cells were also stimulated with a depolarizing solution such as 60 mM KCl able to activate the voltage-gated channels, including the Ca^2+^ ones. As shown in Figure 4F, all compounds led to characteristic kinetics indicating that these cells not only express markers of mature cardiomyocytes, but are also able to respond to stimuli for endogenous GPCRs and channels.

#### 4.2. Neurons

Neurons are another cell type in which Ca2+ plays a fundamental role in signal transduction, particularly in the release of neurotransmitters.

**Figure 5:**
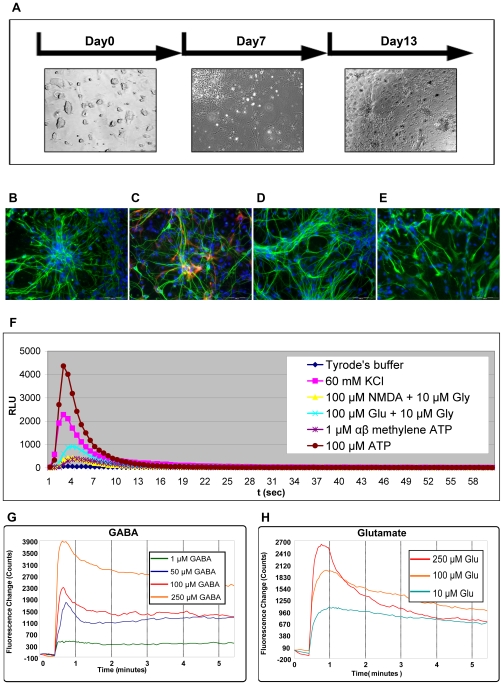
*In vitro* neuronal differentiation protocol on mES/mito c-Photina/29 clone. **A.** Morphological analysis of the cells at different day of the development (contrast phase images). Scale bar −200 µm. **B.−E.** Immunofluorescence analysis with: **B.** Anti-nestin antibody counterstained with Hoechst 33342 dye. Scale bar −100 µm. **C.** Anti-beta III tubulin antibody (green) and anti-GFAP antibody (red) counterstained with Hoechst 33342 dye. Scale bar −100 µm. **D.** Anti-MAP-2 antibody counterstained with Hoechst 33342 dye. Scale bar −100 µm. **E.** Anti-GFAP antibody counterstained with Hoechst 33342 dye. Scale bar −100 µm. **F.** CCD camera-based functional test on *in vitro* differentiated neurons. Tyrode's buffer, 60 mM KCl, 100 µM NMDA+10 µM glycine, 1 µM alpha, beta metylene ATP, and 100 µM ATP responses were measured in 384 MTP on day 13 differentiated neurons. *Lumibox luminometer conditions: high sensitivity, reading time 60 seconds*. **G.-H.** FLIPR^384^ functional test on *in vitro* differentiated neurons at day 13. For FLIPR® analysis the medium was replaced with 25 µL/well of Membrane Potential (MP) fluorescent dye. The plate was then incubated for 30 min at 37°C and for 30 min at room temperature and then 12.5 µL/well of GABA (**G.**) and glutamate (**H.**) solution at different concentration were injected (3X concentrated) and the fluorescence signal was recorded and expressed as RFU (Relative Fluorescence Units). *FLIPR^384^ settings: Exp. Time: 0.3 sec; injection speed: 20 µL/sec; injection height: 50 µL; reading time: 360 seconds*.

For neuronal differentiation, the mES cells were differentiated in monolayer. The presence of cellular processes were visible 4–5 days after plating on gelatin-coated tissue culture dishes, and their length increased over time (Figure 5A). The presence of specific markers in these cells was investigated by immunofluorescence. The nestin staining indicated the presence of neural precursors at day 13 of differentiation (Figure 5B), together with betaIII tubulin and MAP-2 labelling which detected differentiated neurons (Figure 5C–D), as well as staining for GFAP (Figure 5E) which indicated the presence of astrocytes, among the population of differentiated cells.

The functionality of these cells was explored with the Lumibox luminometer. At differentiation day 13, the cells were stimulated by injecting glutamate and NMDA plus glycine, in order to investigate the glutamate receptors, or with ATP and αβ methylene ATP, for purinergic receptors, or depolarized with 60 mM KCl, for the activation of voltage-gated channels, including the Ca^2+^ ones. All stimulations produced the expected response curves (Figure 5F).

To validate the reporter characteristics of c-Photina in this context, we characterized activation of a GqPCR (group I metabotropic glutamate receptor), a Ca^2+^-permeable, ligand-gated ion TRP (Transient Receptor Potential) channel (vanilloid receptor-1), and voltage-gated Ca^2+^ channels, in our neuronally differentiated cells (at differentiation day 13) by comparing the photoprotein-based luminescent read-out to an acetoxymethylester-coupled dye-based (Fluo4NW) fluorescent read-out on the FLIPR^tetra^ instrument (Figure S1). The similar results further confirm the ability of our system to detect both extracellular and intracellular Ca^2+^ influxes.

With this differentiation protocol, we expected to have a high percentage of GABA-ergic and glutamatergic neurons [20]. Since GABA is a chloride channel and glutamate receptors permit passage of not only Ca^2+^ but also Na^+^, we analyzed the cells with the FLIPR^384^ using the voltage-sensitive dye (named Membrane Potential dye) able to detect non Ca^2+^ ionic flux into the cells. At differentiation day 13, after stimulation with GABA and glutamate compounds at various concentrations, we observed a dose-dependent response with both agonists (Figure 5G–H). By using picrotoxin, a specific GABA channel antagonist, we verified that this GABA-mediated signal was mainly due to activation of the GABA channels and not of the GABA transporters (data not shown). Note that the GABA response in Figure 5G is depolarizing. This is consistent with the previous observation that GABAergic reversal potential (E_GABA_) is more liable to depolarize the membrane potential in immature as compared to adult neural cells. This difference is due to a variation of the intracellular Cl^−^ concentration which changes during development, due to the presence of the K^+^-Cl^−^ cotransporter KCC2 [21].

### 5. Systematic Characterization of Ca^2+^ Signals in mES As Compared to Neural Cells

Extrinsic signals from ‘niche’ play an important role in maintenance of multipotency in stem cells. mES cells are pluripotent and model the cell intrinsic response to signals that maintain multipotency. But the cellular pathways known to transduce these signals are few [22], and Ca^2+^ signalling has been only partially characterized in embryonic stem cells [23]. Therefore, we screened undifferentiated and neuronal differentiated cells with an unbiased library of pharmacologically active compounds (LOPAC^1280TM^). This library contains all the major pharmacological target classes, including GPCRs, and ion channels active compounds. We asked which receptors and ions were functional in undifferentiated mES-c-Photina cells, as compared to neural differentiated c-Photina cells. The results were expressed as “percent activity” with respect to ATP (“max” signal for the undifferentiated cells) and glutamate (“max” signal for the differentiated cells). ATP and glutamate were selected as reference compounds, since these agonists show the highest response in the cell populations tested. The data were then analyzed with the two-sample unequal variance, one-tailed t-Student test. All the compounds showing a t-test value less than 0.05 and a percent activity mean value higher than the one showed by the mean of the min signals were selected as positives and included in table 2 (see also Table S1 and Figure S2).

**Table 2:** Active LOPAC1280TM agonist compounds.

Group	Name	Percent Activity Mean ± Standard Deviation
Undifferentiated mES cells	2-Chloroadenosine	57±22.3
	Calcimycin	61±10.4
	2-Chloroadenosine triphosphate tetrasodium	36±10.5
	Histamine dihydrochloride	13±6.1
	Hydrochlorothiazide	34±13.5
	P1,P4-Di(adenosine-5′)tetraphosphate triammonium	24±13.5
	Spiperone hydrochloride	44±18.5
	D-609 potassium	17±0.8
	Thapsigargin	41±12.1
Differentiated mES cells, Day 13	Chelerythrine chloride	15±2.0
	L(−)-Norepinephrine bitartrate	97±32.0
	Acetyl-beta-methylcholine chloride	12±6.3
	6-Fluoronorepinephrine hydrochloride	58±23.4
	(±)-Norepinephrine (+)bitartrate	108±21.7
	Tryptamine hydrochloride	9±4.0
	2-Methylthioadenosine triphosphate tetrasodium	132±23.1
	Calcimycin	41±24.4
	(S)-3,5-Dihydroxyphenylglycine	17±11.5
	(±)-AMPA hydrobromide	21±4.8
	(−)-Epinephrine bitartrate	390±162.8
	2-Chloroadenosine triphosphate tetrasodium	226±3.9
	(−)-alpha-Methylnorepinephrine	57±21.7
	Histamine dihydrochloride	28±15.5
	(+)-cis-Dioxolane iodide	54±41.8
	(±)-Epinephrine hydrochloride	113±8.6
	L-Glutamic acid hydrochloride	16±9.1
	OXA-22 iodide	10±1.3
	N-Methyldopamine hydrochloride	21±14.0
	FPL 64176	166±77.9
	P1,P4-Di(adenosine-5′)tetraphosphate triammonium	16±5.6
	Kainic acid	24±16.2
	2-Methylthioadenosine diphosphate trisodium	122±46.9
	Phenylephrine hydrochloride	17±9.6
	Oxotremorine methiodide	41±16.4
	Spiperone hydrochloride	47±34.9
	Sanguinarine chloride	45±34.0
	(+)-Quisqualic acid	57±38.6

List of compounds active on undifferentiated mES cells (9 compounds) and on neural differentiated mES cells, at day 13 (28 compounds), with the relative percent activity mean ± the standard deviation.

The data revealed very few extrinsically activated Ca^2+^ signalling pathways in the undifferentiated mES cells. The Ca^2+^ related signals that were present are mainly associated with histamine and purinergic compounds, indicating that histamine and purinergic receptors are present. Interestingly, one of these receptors, the adenosine 1, in the P1 purinergic class, has not been previously noted in undifferentiated mES cells. There were a number of additional active compounds able to induce intracellular Ca^2+^ elevations in the neural cells. Most of these substances suggested receptors compatible with the expected prevalence of receptors in neurons. These data offer additional evidence for the robust and appropriate differentiation of the c-Photina neurons.

### 6. Photoprotein Transgenic Mouse

In addition to providing cells differentiated in culture, the mES cells can be exploited to generate a transgenic mouse, which might serve as a direct source of primary cells that could express both the photoprotein transgene, and an endogenous pharmacological target in the native physiological context. The 2 chimeric mice obtained by germline transmission (as described above) were crossed with C57BL/6 female mice and gave rise to agouti progeny. All the litters were genotyped in order to check for the presence of the transgene. As expected, half of the mice born from these crosses were heterozygous for the c-Photina photoprotein gene (32/64) (Table 1). We named these animals PhotoTopo® mice. The heterozygous mice were crossed, in order to obtain a homozygous population. One fourth of the offspring were homozygous and phenotypically normal, demonstrating that the transgene did not disrupt any gene crucial for survival.

### 7. c-Photina Expression and Activity in PhotoTopo

**Figure 6:**
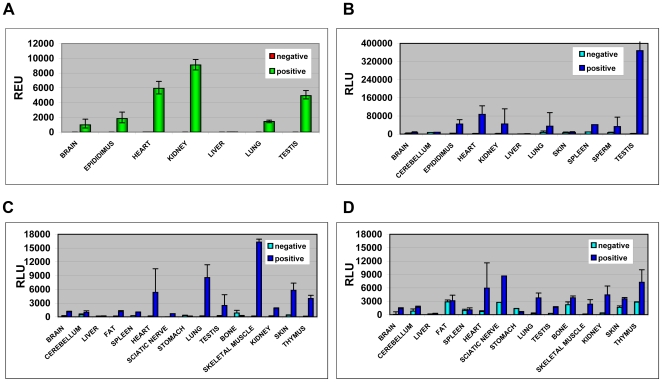
c-Photina tissue localization. **A.** c-Photina expression profile in PhotoTopo animals. 5 mice were sacrificed: 2 negatives and 3 positives and 7 different tissues/organs were isolated and pooled and used for TaqMan® quantitative PCR analysis in triplicates. The data were normalized relative to the amount of 18S rRNA levels and expressed as REU (Relative Expression Units). **B.** c-Photina light activity in PhotoTopo animals. 8 transgenic mice and 6 negative mice from the same litter were sacrificed in 3 different experiments and 11 different tissues/organs were explanted. The cells were incubated for 3 h with 20 µM coelenterazine at room temperature. The photoprotein content was measured injecting a solution of 250 mM CaCl_2_ and 1% Triton X-100. *Lumibox luminometer conditions: high sensitivity, reading time 60 seconds*. **C.-D.** Coelenterazine systemic injection via tail vein in a PhotoTopo mouse and in a negative control. After 3 h, 12 different tissues/organs were explanted from both mice. Half of the material was directly seeded in a white 96 MTP (C.), the other half was further incubated at room temperature for 3 h with 20 µM coelenterazine (**D.**). All the samples were analysed at CCD camera based luminometer for testing the photoprotein content, injecting a solution of 250 mM CaCl_2_ and 1% Triton X-100. *Lumibox luminometer conditions: high sensitivity, reading time 60 seconds*.

To determine the *c-Photina* m-RNA expression profile, we performed a TaqMan® qPCR analysis on transgenic and control samples. The results were normalized to the amount of 18S rRNA level (Figure 6A). Expression was detected in most tissues and at high levels. In order to check for functional c-Photina, 8 transgenic mice containing the *c-Photina* gene and 6 negative mice from the same litter were sacrificed in 3 different experiments. Several tissues were removed from mice, and all samples were incubated in an isotonic solution containing coelenterazine to form the active photoprotein complex. All the isolated tissues were tested in triplicates, injecting a 1% Triton® X-100 plus 250 mM CaCl_2_ solution, in order to discharged all the photoprotein-coelenterazine active complexes into an enriched Ca^2+^ environment (Figure 6B). We found a good correlation between *c-Photina* mRNA expression and light emission across tissues. In addition, light emission remained approximately stable in all expressing tissues of animals of 3, 6, or 10 months old (data not shown).

Furthermore, we investigated the bioavailability of coelenterazine after intravenous systemic injection via the tail vein [24]. After 3 hours, one transgenic and one non-transgenic animal were sacrificed and several tissues/organs were removed. Half of the material was tested immediately with the Lumibox luminometer after cell lysis and injection of a Ca^2+^ solution (Figure 6C). The other half of the material was incubated for another 3 hours with a solution containing coelenterazine, and tested in the same way (Figure 6D). We found that the profile of luminescence across tissues was comparable between intravenous coelenterazine injection-*in vivo* formation of the photoprotein-coelenterazine complex, or after incubation *ex vivo*. The tissue samples with highest luminescent signals were: spleen, heart, lung, testis, kidney, and skeletal muscle (Figure 6).

### 8. Primary Cells Cultured from PhotoTopo

#### 8.1. Aortic endothelial cells

**Figure 7:**
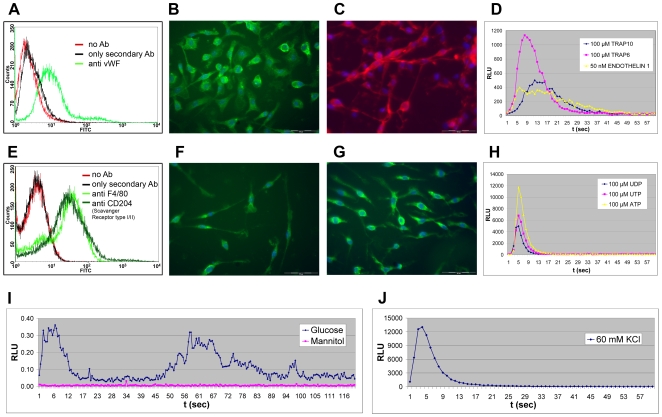
PhotoTopo primary cells. **A.-D.** PhotoTopo derived aortic endothelial cells. **A.** Flow cytometry analysis on aortic endothelial cells isolated from seven animals (4 positives and 3 negatives) with antibody anti-von Willebrand Factor (vWF) and with only secondary antibody or without any antibody as controls. **B.-C.** Immunofluorescence analysis with antibody anti vWF (**B.**), and anti CD31 (**C.**). Scale bar −50 µm. **D.** Lumibox luminometer functional test performed stimulating the aortic endothelial cells with 100 µM TRAP-6 and −10 peptides (agonists for the proteinase-activated receptors) and 50 nM endothelin-1 (agonist for endothelin receptors). *Lumibox luminometer with the following settings: high sensitivity, reading time 60 seconds*. **E.-H.** PhotoTopo derived bone marrow monocyte/macrophage cells. **E.** Flow cytometry analysis on mature cells after 10 days of differentiation obtained from seven animals (4 positives and 3 negatives) with antibody anti-F4/80 and scavenger receptor type III (CD204), and with only secondary antibody or without any antibody as controls. **F.-G.** Immunofluorescence analysis with antibody anti F4/80 (**F.**) and scavenger receptor type III (**G.**). Scale bar -50 µm. **H.** Lumibox luminometer functional test performed using UDP, UTP, and ATP (100 µM) as agonist for purinergic receptors. *Lumibox luminometer conditions: high sensitivity, reading time 60 seconds*. **I.-J.** CCD camera-based luminometer functional test performed on pancreatic islets isolated from transgenic mito c-Photina mice. Pancreatic islets were isolated from 1 positive transgenic mito c-Photina and 1 negative mouse. After an overnight culture, 10 islets/well were put in a white 96 MTP. **I.** Two different wells containing 10 islets each were incubated for 3 h with 10 µM coelenterazine and stimulated respectively with 11 mM glucose or 11 mM mannitol as negative control. *Luminoskan luminometer. Integration time 0.5 sec. Reading time 150 seconds*. **J.** The islets were then stimulated with 60 mM KCl. Light emitted was measured at Lumibox. *Lumibox luminometer conditions: high sensitivity, reading time 60 seconds*.

Aortic endothelial cells are very important cells for cardiovascular diseases, and are difficult to obtain in primary culture. Since Ca2+ has a fundamental role also in these endodermally-derived cells, we decided to isolate these cells. Seven animals (4 positives and 3 negatives) were sacrificed and aortas explanted. After plating on MatrigelTM and culture for 11 days in presence of endothelial cell growth supplement and heparin, we obtained endothelial cells [25]. The presence of these cells was demonstrated by flow cytometry and immunofluorescence analysis using von Willebrand Factor (vWF) and CD31/PECAM-1 markers. The cells were also tested functionally by seeding 50,000 cells/well in a 96 MTP and stimulated with endothelin-1 and TRAP 10 and 6 peptides, agonists for the endothelin receptor and proteinase-activated receptors, respectively, both of which are highly expressed in endothelial cells. As shown in Figure 7A–D activation of both receptors induced a Ca2+ mobilization from internal stores giving rise to typical kinetics of light emission.

#### 8.2. Bone marrow-derived monocytes/macrophages

The PhotoTopo mice are also a source of stem cells and precursors. To verify that the c-Photina was active in hematopoietic monocyte lineage, bone marrow-derived monocyte/macrophage precursors were isolated from the femurs of 4 positive and 3 negative transgenic mice and cultured for 10 days in the presence of M-CSF (Macrophage - Colony Stimulating Factor) [26]. We confirmed the presence of mature macrophages in cell culture, by staining for specific markers F4/80 and scavenger receptor type III (CD204) in flow cytometry and immunofluorescence analysis (Figure 7E–G). Functional studies were performed in these cells by injecting a solution of 100 µM UTP, UDP and ATP in order to stimulate the purinergic receptors. As reported in Figure 7H, all agonists were shown to induce appropriate Ca2+-mediated light emission.

#### 8.3. Micro-organs: beta islets

Next we analysed if c-Photina would trace the glucose-triggered and Ca2+-mediated secretion of insulin in islet cells, representing micro-organs. A pancreatic islet isolation and purification was performed from PhotoTopo animals and from negative controls. Islets were cultured overnight at 37°C, and, the following day transferred to 96 MTP (10 islets/well). After incubation with Krebs-Ringer's buffer in presence of coelenterazine, they were stimulated with 11 mM glucose in order to activate the Ca2+-mediated insulin pathway. As control, mannitol which does not induce the Ca2+-mediated insulin response was injected at the same final concentration in order to maintain the same osmotic concentration (Figure 7I). We observed waves of Ca2+-mediated luminescence, induced only after stimulation with glucose and not with mannitol. The islets were then stimulated with a depolarizing agent (60 mM KCl) which induces a massive Ca2+ influx through the voltage-gated Ca2+ channels and produced robust light emission (Figure 7J).

### 9. Ex Vivo Bioimaging from the PhotoTopo

**Figure 8:**
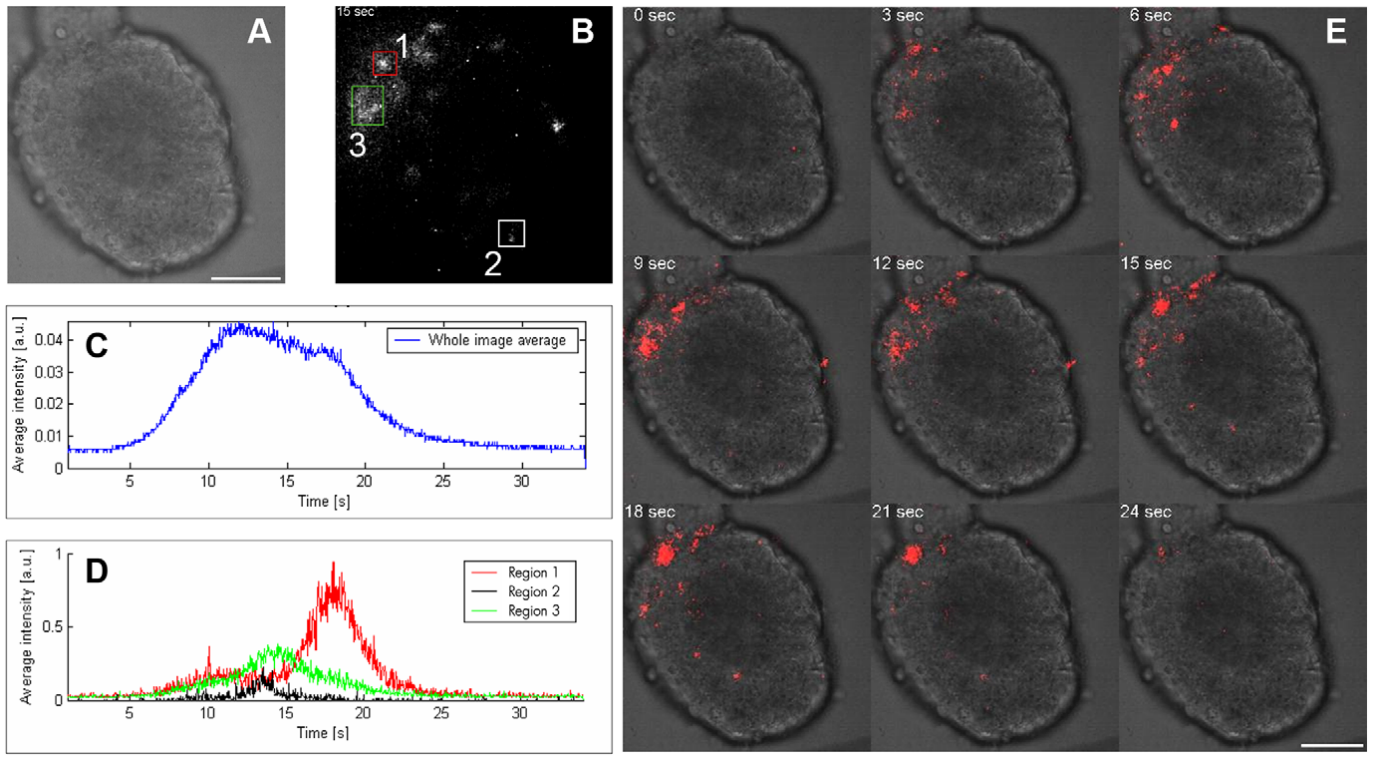
PhotoTopo pancreatic islet bioimaging. **A.-E.** A single islet (**A.** contrast phase) was stimulated with 60 mM KCl in order to activate voltage-gated Ca^2+^ channels. The influx of Ca^2+^ induces an activation of the c-Photina and the emission of light. The light emitted was acquired using an intensified CMOS based camera with 512×512 pixel resolution at 60 frames/sec. The images were then elaborated with ImageJ software and integrated in a total bin (**B.**). With ImageJ is possible to retrieve the kinetics of the whole islet (**C.**) or those of the single regions (**D.**). **E.** The polar injection of the stimulus induces a Ca^2+^ mobilization visible in sequential integrated picture frames (see also Video S3). Scale bar −50 µm.

To further explore the potentiality of c-Photina, we performed luminescence-based bioimaging studies on pancreatic islets. In order to detect topographical light emission in islets, we exploited a microscope-based device, equipped with an intensified CMOS camera (Photron Fastcam). Subsequent to a polarized injection of 60 mM KCl solution, we observed light emission representing Ca^2+^ moving across the entire beta islet (Figure 8A-B-E and Video S3). After acquisition, it was possible to retrieve either the response kinetics recorded from the whole islet (Figure 8C), or define the response kinetics from single areas (Figure 8D).

## Discussion

The generation of a pluripotent embryonic stem cell line containing a Ca^2+^-activated photoprotein offers many opportunities to study Ca^2+^-based signals. In fact, we demonstrated that c-Photina stem cells can be differentiated into two specific cell types and can be used as source of multiple primary-like cells for Ca^2+^ functional studies. Furthermore their pluripotency allowed the generation of transgenic mice, which can be an interesting reserve of cells, such as the adult stem cells (for example haematopoietic stem cells), committed progenitors, and also primary cells containing the photoprotein, for pharmacological or bioimaging studies. Interestingly, the animals can additionally be crossed with other animal models, in order to exploit these possibilities in the context of disease.

Monitoring Ca^2+^ signalling with a Ca^2+^-sensitive reporter gene in mES cells, primary cells, and in a whole animal model, opens many opportunities to understand the development, function, and plasticity of many crucial Ca^2+^-mediated pathways. Several techniques have been described for measuring intracellular Ca^2+^. Patch-clamp and Ca^2+^ selective microelectrodes allow quantitative measurements of Ca^2+^ fluxes in single-cell analysis. These ion-selective microelectrodes (ISMs) are highly sensitive and selective, but suffer from a slow response time, and high levels of noise [27]. Furthermore this technology can be applied only to a restricted number of cells. On the other hand, large populations of cells can be investigated for intracellular Ca^2+^ dynamics with fluorescent probes [28]. In addition to fluorescent dyes there are also genetic tools, which provide fluorescent-based methods for Ca^2+^ monitoring, and are basically divided in two groups. The first category uses the principle of fluorescence resonance energy transfer (FRET) between two variants of the green fluorescent protein (GFP), covalently linked with Ca^2+^ binding proteins like calmodulin [29], [30]. The second category is composed of bioluminescent proteins such as aequorin [31] fused with a GFP molecule. This latter approach is used both in single cells and in transgenic animals [32], [33]. The configuration of the construct with the fusion of the two proteins allows intramolecular chemiluminescence resonance energy transfer (CRET). In fact, after Ca^2+^ binding, aequorin, in presence of coelenterazine, emits a quantum of light that is transferred to GFP, which works as an acceptor and emits green light [32]. The advantage of using CRET approach, instead of using only the aequorin bioluminescent signal, is to overcome the low light quantum yield of the photoprotein. The combination of the two proteins in CRET, in fact, permits detection of the Ca^2+^-mediated signal, even with unavoidable loss of energy during the transfer.

Here we reported the development of a more direct and simpler system to measure efficiently Ca^2+^ movements without any loss of energy during the transfer from the photoprotein to the GFP proteins. To do this we transfected directly only the photoprotein gene in mES cells and then we used these cells to develop a transgenic animal. The choice to use only the bioluminescent reporter gene avoids fusion proteins that could induce, even in presence of tethers, problems of folding and translation. This might be of particular relevance if we consider the larger dimension of the fusion construct compared with the single photoprotein gene. We strategically used the new photoprotein c-Photina and we demonstrated its successful application to detection of Ca^2+^-induced quantum of light using optical systems both in stem cells and in organs from transgenic animals.

An important additional feature of the mitochondrial tagged c-Photina photoprotein is its cellular stability, both in mammalian cells even in absence of a selective pressure, and in an embryonic stem cell line, and in a transgenic mouse model. This is not trivial since from our unpublished observation the expression of many natural and recombinant photoproteins often decreases during time, especially in the absence of selective pressure. The photoprotein was targeted to mitochondria, given the crucial role of Ca^2+^ homeostasis in them. In fact, besides a central function in cell energy metabolism, mitochondria are able to modulate cytosolic Ca^2+^ concentration and participate in Ca^2+^ signalling. Moreover, mitochondrial Ca^2+^ uptake is a phenomenon involved not only after Ca^2+^ release from intracellular stores, as happens after stimulation of GqPCRs, but also after Ca^2+^ influx through specific Ca^2+^ channels from extracellular space [34], [35],(for review see [36], [37]). Accordingly, our mitochondrial tagged photoprotein actually efficiently recorded cytoplasmic Ca^2+^ variations, after activation of both Ca^2+^ channels, such as the voltage-gated Ca^2+^ channels, P2X purinergic channels, and TRP channels such as the vanilloid receptors.

We demonstrated that the presence of the photoprotein does not interfere with the pluripotency of mES cells. In fact, these cells still express stemness markers like oct 3/4 transcription factor and the SSEA-1 surface antigen and possess alkaline phosphatase activities [16]–[18]. Furthermore, the c-Photina mES cells, when injected into the blastocyst of a recipient surrogate mother, gave rise to germline transmission with a high efficiency (Table 1). The pluripotency of the c-Photina stem cells was also confirmed by the ability of the c-Photina mES cells to differentiate *in vitro* into cell types derived from different germ layers, such as cardiomyocytes and neurons.

The possibility to generate primary cells such as cardiomyocytes and neurons containing a bioluminescent system for monitoring Ca^2+^ movements finds many applications. Since, Ca^2+^ has a crucial role, for example, in controlling cardiac rhythm, one could investigate Ca^2+^ behaviour in many models of different cardiac diseases. Also in neurons it could be interesting to visualize Ca^2+^ movements, in normal development or during neurodegeneration, or address synaptic function. We optimized the differentiation protocols also in miniaturized formats, like the 96 and the 384 MTP, *sine qua non* condition for the feasibility of their usage in high throughput screening. Cells differentiated in these formats showed all the characteristics of primary-like cells, such as the expression of cell-specific targets (demonstrated by immunofluorescence analysis) or the ability to spontaneously pulse or to respond to agonist for receptors highly expressed in these cells. Moreover, the differentiation processes were shown not to interfere with the expression of the reporter gene. The functional presence of the photoprotein in the differentiated cells was demonstrated by the ability of the cells to respond to different stimuli, inducing Ca^2+^ movements, confirming their utilization suitable for the development of cell-based assays.

This approach was exploited to address which Ca^2+^ receptors or channels might have a functional role in mouse embryonic stem cells. We screened an unbiased library of 1280 pharmacological active compound (LOPAC^1280™^) modulating all of the major classes of important receptors and channels. This signalomic approach allowed high throughput identification of all cell surface receptors and channels whose activation induce a variation of intracellular Ca^2+^ in undifferentiated mES cells. As a positive control for known Ca^2+^ signalling a parallel approach was performed on neuronally-differentiated cells. The results indicated sparse activation of Ca^2+^ response in the undifferentiated mES cells. The only classes of receptors that were shown to be activated were the one of histamine-1 receptor (H_1_) and those of purinergic receptors, both already described in literature [15], [23], [38]. The presence of the H_1_ receptor in these cells suggests a role of histamine in early mammalian development. Furthermore, histamine was shown to have a role in regulating neural stem cells proliferation and the expression of the H_1_ receptor was shown to favour neuronal fate [39]. Also the presence of P2 purinergic receptors was already described in mouse embryonic stem cells. In particular it was proposed a role of extracellular ATP in stimulating mouse embryonic stem cell proliferation [38]. Here the authors revealed the presence of P1 purinergic receptors too by the functional response to 2-chloroadenosine only in undifferentiated and not in neuronally-differentiated cells. Its activation suggests that the role of the P1 receptor in pluripotent mouse embryonic stem cells should be further investigated.

Thanks to germline transmission of c-Photina mES cells, a transgenic animal containing the c-Photina photoprotein (PhotoTopo) was derived. The cells cultured from photoprotein transgenic animals can be used as positive controls for the “primary-like” cells obtained after differentiation of mES cells, and directly as primary cells, for a pharmacological screening process *per se*. As proof of principle, we isolated from PhotoTopo animals primary endothelial cells and hematopoietic precursors, which we differentiated into bone marrow-derived monocytes/macrophages. We demonstrated that these cells contain the photoprotein and that they can be used in miniaturized format for functional assays. Moreover, the organism-wide expression of the transgene in PhotoTopo mice (Figure 6A–B) suggests the availability of a larger spectrum of useful primary cells. Interestingly, the expression of the reporter gene was shown not to decrease with animal age, further confirming the stability of this photoprotein and indicating that the transgene is not inserted in a position subject to chromatin inactivation over the course of time.

The c-Photina transgenic mouse, can be used in combination with optical microscope systems, like CCD cameras, which are able to detect in real time light emission of bioluminescent reporter within the animal's cells. This application opens the possibility to charge the photoprotein by systemic injection of coelenterazine, representing a suitable model for monitoring modulation of intracellular Ca^2+^ levels, and for the generation of *in vivo* bioluminescence imaging (BLI) based studies. As proof of principle we isolated PhotoTopo pancreatic islets since they are a perfect source of material for studying complex Ca^2+^ exchanges, occurring between different cell types. In fact they are multicellular structures, in which Ca^2+^ plays a fundamental role in insulin secretion. Actually, the entrance of glucose through the type 2 glucose transporters induces the activation of voltage-gated Ca^2+^ channels. The consequential entry of Ca^2+^ ions from the extracellular space induces insulin release from insulin-storing granules exocytosis. We demonstrated the feasibility of Ca^2+^ movement observation in PhotoTopo islets, after a glucose and a depolarizing stimulus, through the photoprotein activation, not only by a CCD camera-based luminometer but also by using an intensified CMOS-based camera with 512×512 pixel resolution. The potentiality to study Ca^2+^ movements in whole islets and within single cells opens very interesting potential applications for diabetes research.

## Materials and Methods

### c-Photina Generation

The *c-Photina* gene (mutant 12, Patent Application EP06000171) was obtained by random mutagenesis from the gene *Clytin* (GenBank accession number Q08121) using the GeneMorph II Random Mutagenesis kit (Stratagene, La Jolla, CA, USA) following the supplier's instructions with the following primers:

Upper: GATGACGACGACAAG-ATGGCCGACACCGCCAG

Lower: GAGGAGAAGCCCGGT-TTATCAAGGACACGAAGT.

### mES Cell Culture

TBV2 (129S2/SvPas) mouse embryonic stem cells [40] were cultured in the undifferentiated state on a monolayer of Mitomycin C treated mouse embryonic fibroblasts in the presence of leukemia-inhibiting factor (LIF) (Chemicon) [41].

### Photoprotein mES Cell Clone Generation and Selection

The *c-Photina* gene was cloned into the pcDNA3.1+ vector (Invitrogen) downstream the mitochondrial tag (mito) of the human *Cytochrome C Oxydase*, subunit VIII [14].

7×10^6^ mES cells were electroporated using 30 µg of the *mito c-Photina* DNA, linearized with BglII (New England Biolabs). Positive clones were selected with 200 µg/mL G418 (geneticin, SIGMA) [40]. Four hours before the test the medium was replaced with 50 µl/well of Tyrode's buffer (130 mM NaCl, 5 mM KCl, 2 mM CaCl_2_, 1 mM MgCl_2_, 5 mM NaHC0_3_ and 20 mM HEPES, pH 7.4, 2 mM Ca^2+^) and 10 µM coelenterazine, in the dark, and incubated at 37°C in a humidified atmosphere with 5% CO_2_ in order to reconstitute the active photoprotein. The number of photons emitted after injection of the different ligands for 60 seconds was measured on the Lumibox CCD camera-based luminescence detector designed and built by Bayer Technologies GmbH (Wuppertal, Germany), and expressed as RLU (Relative Luminescence Units).

DNA from mES cells plated on gelatin-coated dishes was extracted with standard methods [42]. 10 µg of ES genomic DNA of ES/mito c-Photina cells was digested with the restriction enzymes, HindIII, XbaI, BamHI, HindIII/XbaI (New England Biolabs), transferred to a positively-charged nylon membrane (Roche) for Southern blot analysis. The [^32^P]dCTP–labelled *c-Photina* coding sequence [42] was used as the probe. The Southern blot analysis was performed by digesting the genomic DNA with restriction enzymes which cut only once in the transfected vector (to discriminate concatamers).

### Quantitative PCR – Sybr Green®

QPCR (Quantitative Polymerase Chain Reaction) was performed using approximately 3 ng of DNA per reaction with the “Platinum® SYBR Green® QPCR SuperMix UDG” protocol (Invitrogen). The primers used were designed on *c-Photina* (cPH), *neomycin* (neo) and *gusB* gene:

cPH-for: CACCAAGTGTGCGTGGAGG; cPH-rev: GCGATCTCCTTGCCGTACTC;

neo-for: CACGTACTCGGATGGAAGCC; neo-rev: CCCTGATGCTCTTCGTCCAG;

gusB-for:GGAGGTGATTCAGCCACAGC; gusB-rev: TCGGCTTCTGATGCGTCTTA.

All QPCR experiments were run on an ABI Prism 7700 Sequence Detector (Applied Biosystems). To calculate the number of copies we used the following formula:
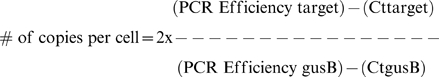



### Production of Transgenic Mice and Germline Transmission Test

All experiments involving animals were performed in strict accord with experimental protocols approved by the San Raffaele Institutional Animal Care and Use Committee and with Italian National Health Ministry regulations. ES 29 clone was injected into C57BL/6 blastocysts to generate chimeric mice (estimated by hair color and tail genomic DNA analysis) [40]. Two chimerae were mated with C57BL/6 mice to obtain germline transmission of the mutation (see Table 1). Genotype analysis was performed on genomic DNA prepared from tail snips using the primer pair:

Primer for: AACTTCGACAACCCCAAGTG,

Primer rev: TGTCGAAGATGTCGAACACG, that recognize the *c-Photina* coding sequence.

### Cardiomyocyte Differentiation Protocol with the Embryoid Bodies (EBs) Formation Step

The cardiomyocyte differentiation protocol was obtained using hanging drops procedure, using 300 cells/drop for 2 days then putting the cells in suspension into bacterial grade dishes for other 3 days in DMEM medium plus 15% fetal calf serum (FCS) (Invitrogen), as described [19]. EBs were dissociated at differentiation day 12 with Accutase™ (Chemicon), plated in 384 MTP plates and tested 48 h after seeding at the Lumibox luminometer. Four hours before the test the medium was replaced with 25 µL/well of Tyrode's buffer and 10 µM coelenterazine, in the dark, and incubated at 37°C in a humidified atmosphere with 5% CO_2_.

### Neuronal Differentiation Protocol

The mES cells are seeded at 1,500 cells/cm^2^ on gelatin-coated dishes in Knock Out-DMEM at 15% KSR (Knock-out Serum Replacement) (Invitrogen), ), at day 7 they were collected with 0.05% trypsin/EDTA solution and replated at 5,000 c/w in gelatin-coated 384 MTP dishes for in total 13–14 days with medium changing every two-three days [20]. The luminescent test was performed using the Lumibox luminometer, and incubating four hours before the differentiated cells with 25 µL/well of Tyrode's buffer and 10 µM coelenterazine, in the dark at 37°C in a humidified atmosphere with 5% CO_2_.

For the fluorescent test with the Membrane Potential dye (Molecular Devices) the differentiated cells were incubated in 25 µl/well of the dye solubilised in Tyrode's buffer for 30 min at 37°C plus 30 min at room temperature. The fluorescence signals were recorded for 250 sec and expressed as RFU (Relative Fluorescence Units) at FLIPR^384^® fluorescent reader. GABA and glutamate solution ligands were injected 3X concentrated (12.5 µL/well) at different concentration.

For the comparison between luminescent and fluorescent read-outs the cells were or incubated with with 40 µL/well of Tyrode's buffer and 10 µM coelenterazine four hours before the test or with 40 µl/well of the Fluo4NW dye (Molecular Devices) solubilised in Tyrode's buffer for 60 min at 37°C. The signals were recorded at FLIPR^tetra^® reader, for 220 sec for fluorescence and 60 sec for luminescence read-out.

### Immunofluorescence Analysis

The mES cells were fixed with 4% paraformaldehyde (PFA, MERCK, Whitehouse Station, NJ, USA) solution and simultaneously blocked and permeabilized with 10% normal goat serum (Chemicon)/0.2% Triton X-100 in 1X PBS.

The different antibodies were incubated in 10% normal goat serum 0.1% Triton X-100 in 1X PBS.

The primary antibodies used were: mouse anti-oct 3/4 (C-10) (Santa Cruz Biotechnology), mouse anti SSEA-1 (Santa Cruz Biotechnology), rabbit anti myosin heavy chain (MHC) (H-300) (Santa Cruz Biotechnology), rabbit anti-GATA-4 (H-112) (Santa Cruz Biotechnology), mouse anti-sarcomeric alpha-actinin (EA-53) (SIGMA), rabbit anti-microtubule-associated protein 2 (MAP2) (Chemicon), rabbit anti-beta III tubulin (Chemicon), rabbit anti glial fibrillary acidic protein (GFAP) (Dako), and mouse anti-nestin (Rat-401) (Chemicon).

The secondary antibodies used were the fluoresceinated anti-mouse (Chemicon), alone or in combination to the anti-rabbit IgG/IgM rhodamine-conjugated (Millipore), or anti rabbit FITC-conjugated (Chemicon) secondary antibody in 10% normal goat serum/0.1% Triton X-100 in 1X PBS.

The Hoechst 33342 dye (Invitrogen) (2 µg/mL final concentration) was incubated for 5 min at room temperature.

Images were acquired by using either an Olympus IX51 microscope equipped with a F-View II camera and the dedicated software cell-F (Olympus) or an Olympus IX70 microscope coupled to a Leica digital camera with a customized acquisition system. In some cases the brightness and/or the contrast were modified in order to reduce the background signal deriving from the white wall 384 plates used for the experiments (Matrix-Thermo Scientific 384 well plates-polystyrene white/clear).

### Alkaline Phosphatase Staining

The alkaline Phosphatase activity was measured with the ELF® Phosphatase staining kit (ATCC) following manufacturer's instructions.

### Taqman® PCR

Total RNA was isolated by TRIzol® (Gibco/BRL, Gaithersburg, MD). Reverse transcription-PCR (RT-PCR) was performed with the Invitrogen Superscript II RT-PCR kit (Invitrogen), as recommended by the manufacturer.

FAM- and MGBNFQ (Minor Groove Binder/Non-Fluorescent Quencher) -dual-labelled TaqMan® “MGB” probes were used as a quality standard. The primers and probe sequences are: Forward primer: GCGCCAAGATCCATTCGTT; Reverse primer: GAACATGAACTTGTGCCGGTT; TAQMAN Probe: CCATGGCCGACACC.

Total cDNA content normalized by simultaneous quantification (by multiplex PCR) of the 18S ribosomal RNA. All experiments were performed on an ABI Prism 7900HT Sequence Detection System (Applied Biosystems), using the comparative C_T_ method [43]. The relative expression units (REU) were calculated as: 1 REU = 2̂-(average Ct Target - average Ct 18S) * 10̂7. The range of variation was determined by evaluating the expression: {2̂-[(Ct_cPh_-Ct_18S_ ± standard deviation]}10̂7, where the standard deviation is calculated as √(σ_cPh_+σ_18S_).

### LOPAC^1280™^ Screening

The 1280 compounds of LOPAC^1280™^ library were reformatted in 384 MTP and diluted at 50 µM in tyrode plus 2.5% DMSO (5X concentrated), the undifferentiated mES cells were seeded in triplicate on 384 gelatin-coated plates 24 h before the tests at a concentration of 20,000 cells/well.

Neural differentiated mES cells were seeded in quadruplicate at a concentration of 5,000 cells/well 6 days before the test (done at day 13 of differentiation).

Prior to the test, all the samples were incubated in Tyrode's buffer containing 10 µM coelenterazine for 3 h.

The LOPAC library was used at the final concentration of 10 µM. Negative control wells contained Tyrode's buffer plus 2.5% DMSO (that is the same concentration as test wells) (defined as “min” signal). The test was performed on the FLIPR^tetra^® instrument.

Spotfire Decision Site® version 9.0 was used for analysis and curve-fitting of the results obtained from the activity determination experiments. The results were expressed as “percent activity” with respect to ATP (“max” signal for the undifferentiated cells; final test concentration: 100 µM) and glutamate (“max” signal for the differentiated cells; final test concentration: 100 µM). ATP and glutamate were selected as reference compounds, since these agonists show the highest response in the cell populations tested.

For differentiated cells, percent activity was computed based upon the median response value of min signal wells and glutamate wells on each plate. Then the percent activity mean and standard deviation for the quadruplicate wells were computed for each compound. For undifferentiated mES cells, percent activity was computed based upon the median response value of the test wells and ATP wells on each plate. Then the percent activity mean and standard deviation for the triplicate wells were computed for each compound.

The large symbols in Figure S2 indicate that the %Activity was statistically significant based upon a t-test, in comparison to the percent activity of min signal wells (taking all of the min signal wells as a large group). The t-test was performed using Excel, two-sample unequal variance (heteroscedastic), two-tailed. The logic used to define a “significant” activity was:
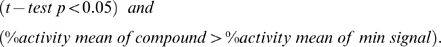



### PhotoTopo CCD Camera Functional Tests

Mice were perfused with a physiological solution and tissues were harvested and incubated in a reaction solution containing 20 mM Tris-HCl pH 7.5, 150 mM NaCl, 5 mM DTT, 1 mM EDTA, 0.1% BSA, 20 µM coelenterazine plus protease inhibitor cocktails (Roche), for 3 h at room temperature. Luminescence was determined on the Lumibox, after injection of a solution of Triton X-100 and 250 mM CaCl_2_.

300 µl of coelenterazine (373 µM coelenterazine, 3.3% DMSO, 990 nM glutathione in physiological solution or 2.8 mg of coelenterazine/kg), was injected via the tail vein. Tissues were explanted, triturated with a scissors, and divided into two batches. One part was put in the reaction solution (20 mM Tris-HCl pH 7.5, 150 mM NaCl, 5 mM DTT, 1 mM EDTA, 0.1% BSA, plus protease inhibitor cocktails) without coelenterazine and tested immediately on the Lumibox; the other part was incubated with the same reaction solution in presence of 20 µM coelenterazine for 3 h at room temperature before the Lumibox luminometer test, after injection of a solution of Triton X-100 and 250 mM CaCl_2_.

### Flow Cytometry

Cells were resuspended in 100 µL of a blocking solution containing 4% FCS serum, 1 mM EDTA in PBS at a cell concentration of 1×10^6^ cells/mL for 10 min, then incubated with 5 µg/10^6^ cells of the primary antibodies for other 30 minutes.

Cells were washed twice with blocking buffer. After 30 min of incubation with the secondary antibody and another 2 washes, cells were resuspended at 10^6^/mL in blocking buffer.

Cell acquisition was performed with FACSort Becton Dickinson in a region (R1) defined gating out only debris by forward and side scatter characteristics. 40,000 gated events were analysed with CellQuest software (BD).

The list of the antibodies used is:

- Typeface="12";-Rabbit anti-human von Willebrand Factor (DAKO),
- Typeface="12";-Goat anti-mouse CD31/PECAM-1 (R&D systems),
- Typeface="12";-Rat anti-mouse CD204 (AbD Serotec),
- Typeface="12";-Rat anti-mouse F4/80 (AbD Serotech),
- Typeface="12";-Goat anti-mouse IgG & IgM, (H+L) FITC conjugated (Chemicon),
- Typeface="12";-Goat anti-rabbit IgG, (H+L) FITC conjugated (Chemicon),
- Typeface="12";-Goat anti-rat IgG, FITC conjugated (AbD, Serotech),
- Typeface="12";-Chicken anti goat IgG (H+L), FITC conjugated (Chemicon).

### PhotoTopo Endothelial Cell Preparation

Endothelial cells were isolated from mouse aorta. The aorta was removed from anesthetized and heparinised mice, and after cleaning was placed (with the intima side down) on Matrigel™ (BD)-coated plates in DMEM medium plus 10% FCS, glutamine, not essential amino acids, and 75 µg/mL endothelial cell growth supplement (ECGS, Sigma). See Suh *et al.*, 1999 [25] for extensive details.

### PhotoTopo Bone Marrow Derived Monocytes/Macrophages Preparation

Mice were sacrificed in order to isolate the bone marrow. The haematopoietic precursors were isolated from bone marrow flushed from femurs and differentiated *in vitro* into macrophages as described [26].

### PhotoTopo Beta Islet Isolation and Culture

Pancreatic islets were isolated from mice (nine weeks old, 20–22 g) by a collagenase digestion method [44]. Briefly, 2 mL of cold Hank's buffer/collagenase type V solution (1 mg/mL; Sigma, St Louis, MS, USA) was infused into the pancreatic duct *in situ*, and the removed pancreas was digested at 37°C for 15 min. Islets were purified on a discontinuous Ficoll gradient (Sigma). The islets (250 islets/mL) were cultured free-floating (37°C, 5% CO_2_) in medium RPMI 1640 (Bio-Whittaker, Walkersville, MD, USA) supplemented with L-glutamine (Sigma), penicillin-streptomycin (1,000 U/mL-10 mg/mL; Sigma) and 10% FCS (HyClone, Celbio, Logan, UT, USA) for 20–24 h before the utilization. Islet purity was >90%.

Ten mito c-Photina transgenic mice islets/well were put in a white 96 MTP and incubated in Krebs-Ringer's solution (125 mM NaCl, 5 mM KCl, 1.2 mM MgSO_4_, 1.2 mM KH_2_PO_4_, 2 mM CaCl_2_, 25 mM HEPES pH 7.4, 0.1% BSA and 3 mM glucose) with 10 µM coelenterazine for 4 h at 37°C. The islets Ca^2+^ kinetic responses were measured at the Luminoskan Ascent (Labsystems) luminometer after stimulation with a glucose stimulus (11 mM), or with mannitol (11 mM), as the negative control. The glucose concentration was then normalized to 3 mM and the islets were then stimulated with a depolarizing stimulus (40 mM KCl) on the Lumibox. The total photoprotein content in the islets was measured after cell were lysed with a Triton X-100-based buffer.

### PhotoTopo Bioimaging

Islets were isolated as described above and seeded 30 islets/35 mm Matrigel^TM^ (BD)-coated glass for 4 h at 37°C in Krebs-Ringer's solution containing 10 µM coelenterazine. Islets were then stimulated with a depolarizing stimulus (60 mM KCl) and light recorded with a set-up based on an Axiovert 200 inverted epifluorescence microscope (Zeiss, Oberkochen, Germany) positioned over an anti-vibration table and equipped with a 40×/1.3NA EC Plan-Neofluar oil immersion objective lens. The light detector was the CMOS technology-based camera Fastcam (Photron, Tokyo Japan).

## Supporting Information

Video S1 Pulsating cardiomyocytes at differentiation day 15. Embryoid body containing a spontaneous beating area of cardiomyocytes obtained 15 days after differentiation of mES c-Photina cells.
(8.51 MB AVI)

Video S2 Pulsating cardiomyocytes at differentiation day 20. Embryoid body containing a spontaneous beating area of cardiomyocytes obtained 20 days after differentiation of mES c-Photina cells.
(80.19 MB AVI)

Video S3 Luminescence-based bioimaging studies on pancreatic islets. Ca^2+^-mediated light release obtained after stimulation of a PhotoTopo beta islet with a depolarizing stimulus (60 mM KCl). The light was acquired with a microscope based device, equipped with an intensified CMOS camera (Photron Fastcam).
(8.92 MB AVI)

Figure S1 Comparison of c-Photina and Fluo4NW readout systems in differentiated neurons (day 13). A–B. Example of responses after activation of a GqPCR, such as Group I metabotropic glutamate receptor, measured at FLIPR^tetra^ instrument with c-Photina (A.) or Fluo4NW fluorescent dye (B.). C–D. Example of responses after activation of a Ca^2+^-permeable, ligand-gated ion TRP (Transient Receptor Potential) channel, such as the Vanilloid Receptor-1 (VR-1), measured at FLIPR^tetra^ instrument with c-Photina (C.) or Fluo4NW fluorescent dye (D.). E–F. Example of responses after activation of voltage-gated Ca^2+^ channels, measured at FLIPR^tetra^ instrument with c-Photina (E.) or Fluo4NW fluorescent dye (F.). *FLIPR^tetra^ settings for luminescence: Integr. time: 1 sec; exp. time: 0.90 sec; injection speed: 20 µL/sec; injection height: 35 µL; reading time: 60 seconds. FLIPR^tetra^ settings for fluorescence: Integr. time: 1 sec; exp. time: 0.53 sec; injection speed: 20 µL/sec; injection height: 35 µL; reading time: 220 seconds.*
(1.55 MB TIF)

Figure S2 LOPAC^1280™^ screening schematic representation. For differentiated (Day 13) cells, Percent Activity is computed based upon the median response value of Min Signal wells and Glutamate (as Max Signal) wells on each plate. For undifferentiated cells, Percent Activity is computed based upon the median response value of the Min Signal wells and ATP (as Max Signal) wells on each plate. The large symbols (square) indicate that the %Activity is statistically significant based upon a t-test, in comparison to the %Activity of Min Signal wells (taking all of the Min Signal wells for each Day as a large group). The t-test performed was the “Two-sample unequal variance, one-tailed”. The threshold lines indicated the percent activity mean ± the standard deviation.
(2.45 MB TIF)

Table S1 Active LOPAC^1280™^ compound description list. List of all the active compounds retrived after screening of undifferentiated and neuronal differentiated cells (day 13) with an unbiased library of pharmacologically active compounds (LOPAC^1280™^). For all the compounds is indicated their complete name, the class, the action, the selectivity and the description (information provided directly by SIGMA LOPAC).
(0.11 MB DOC)
