# Supplementary figures and images for: A Photoprotein in Mouse Embryonic Stem Cells Measures Ca2+ Mobilization in Cells and in Animals
Source: PLoS One. 2010 Jan 27;5(1):e8882. doi: 10.1371/journal.pone.0008882 (PMC2811732; doi:10.1371/journal.pone.0008882)

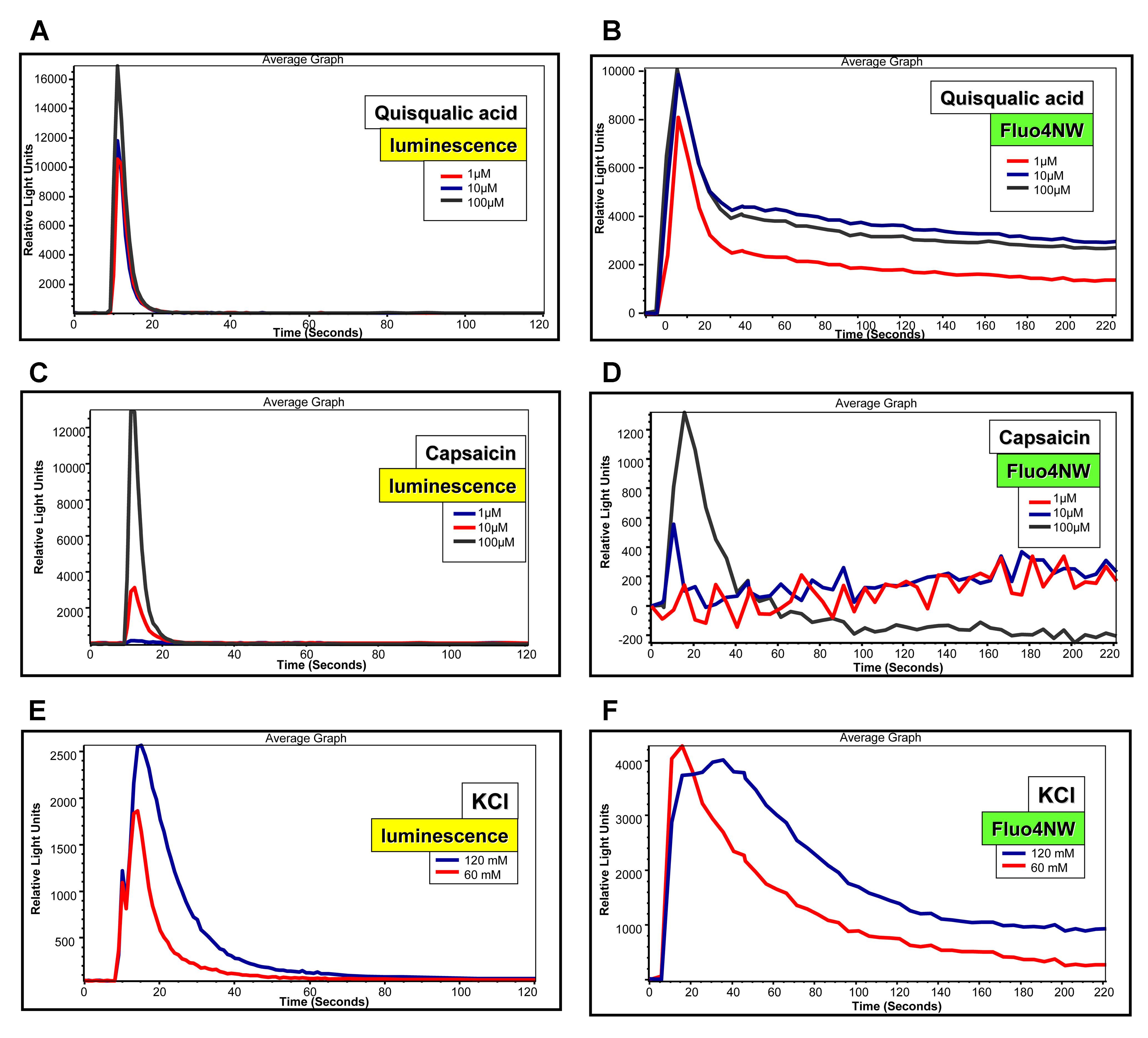

Supplement: Figure S1 — Comparison of c-Photina and Fluo4NW readout systems in differentiated neurons (day 13). A–B. Example of responses after activation of a GqPCR, such as Group I metabotropic glutamate receptor, measured at FLIPRtetra instrument with c-Photina (A.) or Fluo4NW fluorescent dye (B.). C–D. Example of responses after activation of a Ca2+-permeable, ligand-gated ion TRP (Transient Receptor Potential) channel, such as the Vanilloid Receptor-1 (VR-1), measured at FLIPRtetra instrument with c-Photina (C.) or Fluo4NW fluorescent dye (D.). E–F. Example of responses after activation of voltage-gated Ca2+ channels, measured at FLIPRtetra instrument with c-Photina (E.) or Fluo4NW fluorescent dye (F.). FLIPRtetra settings for luminescence: Integr. time: 1 sec; exp. time: 0.90 sec; injection speed: 20 µL/sec; injection height: 35 µL; reading time: 60 seconds. FLIPRtetra settings for fluorescence: Integr. time: 1 sec; exp. time: 0.53 sec; injection speed: 20 µL/sec; injection height: 35 µL; reading time: 220 seconds. (1.55 MB TIF) [file pone.0008882.s004.tif]

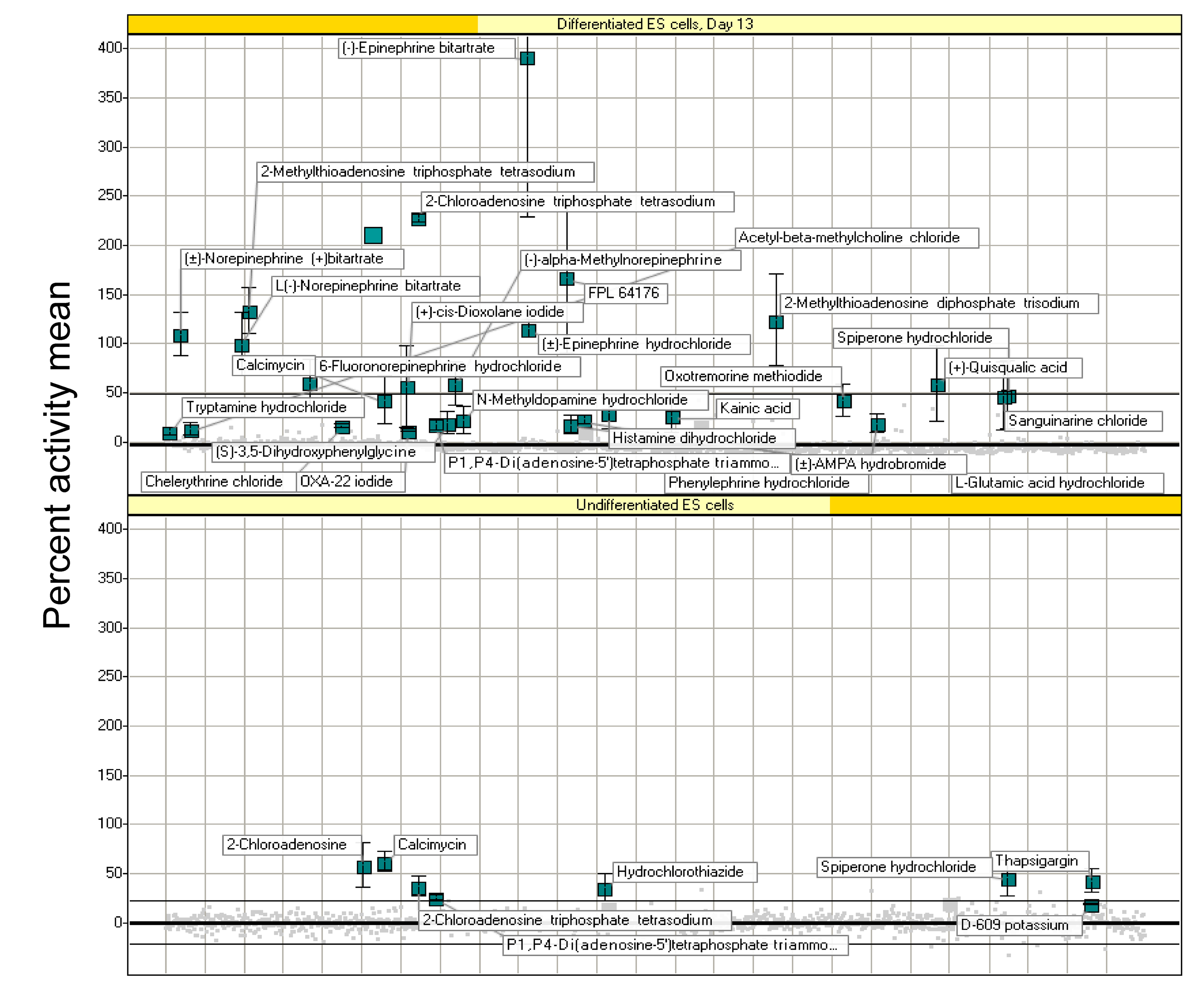

Supplement: Figure S2 — LOPAC1280™ screening schematic representation. For differentiated (Day 13) cells, Percent Activity is computed based upon the median response value of Min Signal wells and Glutamate (as Max Signal) wells on each plate. For undifferentiated cells, Percent Activity is computed based upon the median response value of the Min Signal wells and ATP (as Max Signal) wells on each plate. The large symbols (square) indicate that the %Activity is statistically significant based upon a t-test, in comparison to the %Activity of Min Signal wells (taking all of the Min Signal wells for each Day as a large group). The t-test performed was the “Two-sample unequal variance, one-tailed”. The threshold lines indicated the percent activity mean ± the standard deviation. (2.45 MB TIF) [file pone.0008882.s005.tif]
