# Supplementary material for: A Photoprotein in Mouse Embryonic Stem Cells Measures Ca2+ Mobilization in Cells and in Animals
Source: PLoS One. 2010 Jan 27;5(1):e8882. doi: 10.1371/journal.pone.0008882 (PMC2811732; doi:10.1371/journal.pone.0008882)
Supplement: Table S1 — Active LOPAC1280™ compound description list. List of all the active compounds retrived after screening of undifferentiated and neuronal differentiated cells (day 13) with an unbiased library of pharmacologically active compounds (LOPAC1280™). For all the compounds is indicated their complete name, the class, the action, the selectivity and the description (information provided directly by SIGMA LOPAC). (0.11 MB DOC) [file pone.0008882.s006.doc]

| **Group** | **Name** | **Class** | **Enzyme** | **Action** | **Selectivity** | **Description** |
| --- | --- | --- | --- | --- | --- | --- |
| Undifferentiated mES cells | 2-Chloroadenosine | Adenosine |  | Agonist | A1 > A2 | Adenosine receptor agonist with selectivity for A1 over A2 |
|  | Calcimycin | Intracellular Calcium |  |  | Ca2+ | Ca^2+^ ionophore used to potentiate responses to NMDA, but not quisqualate glutamate receptors |
|  | 2-Chloroadenosine triphosphate tetrasodium | P2 Receptor |  | Agonist | P2Y | P2Y receptor agonist |
|  | Histamine dihydrochloride | Histamine |  | Agonist |  | Endogenous neurotransmitter |
|  | Hydrochlorothiazide | Enzyme |  | Inhibitor | Carbonic anhydrase | Carbonic anhydrase inhibitor; diuretic |
|  | P1,P4-Di(adenosine-5')tetraphosphate triammonium |  |  | Inhibitor |  | Dinucleoside polyphosphate that competitively inhibits ADP-induced platelet aggregation |
|  | Spiperone hydrochloride | Dopamine |  | Antagonist | D2 | Selective D2 dopamine receptor antagonist. |
|  | D-609 potassium | Lipid | Enzyme | Inhibitor | PIPLC | Xanthogenate derivative with in vitro anti-tumor and anti-HIV-1 activity; inhibits phospholipase D and phosphatidylcholine phospholipase C (PIPLC) |
|  | Ropinirole hydrochloride | Dopamine |  | Agonist | D2 | Selective D2 dopamine receptor agonist |
|  | Thapsigargin | Intracellular Calcium | Enzyme | Releaser |  | Potent, cell-permeable, IP3-independent intracellular calcium releaser |
| Differentiated mES cells, Day 13 | Chelerythrine chloride | 1,2-Dimethoxy-N-methyl(1,3)benzodioxolo(5,6-c)phenanthridinium chloride | Enzyme | Inhibitor | PKC | PKC inhibitor; affects translocation of PKC from cytosol to plasma membrane |
|  | L(-)-Norepinephrine bitartrate | (-)-Arterenol bitartrate; Noradrenaline bitartrate |  | Agonist | alpha, beta1 | Adrenergic neurotransmitter; vasoconstrictor |
|  | Acetyl-beta-methylcholine chloride | Methacholine chloride |  | Agonist | M1 | M1 muscarinic acetylcholine receptor agonist |
|  | 6-Fluoronorepinephrine hydrochloride | Methacholine chloride |  | Agonist | M1 | M1 muscarinic acetylcholine receptor agonist |
|  | (±)-Norepinephrine (+)bitartrate | (±)-Arterenol (+)bitartrate; (±)-Noradrenalin (+)bitartrate |  | Agonist |  | Adrenergic neurotransmitter |
|  | Tryptamine hydrochloride | 3-(2-Aminoethyl)indole hydrochloride |  | Ligand |  | Serotonin receptor ligand |
|  | 2-Methylthioadenosine triphosphate tetrasodium | 2-Methylthio ATP tetrasodium |  | Agonist | P2Y | P2Y receptor agonist |
|  | Calcimycin | A23187; Calcium ionophore A23187 |  |  | Ca2+ | Ca^2+^ ionophore used to potentiate responses to NMDA, but not quisqualate glutamate receptors |
|  | (S)-3,5-Dihydroxyphenylglycine | S-DHPG |  | Agonist | mGluR1 | Group I metabotropic glutamate receptor (mGluR1) agonist |
|  | (±)-AMPA hydrobromide | (±)-alpha-Amino-3-hydroxy-5-methylisoxazole-4-propionic acid hydrobromide |  | Agonist | AMPA/kainate | Potent excitatory amino acid that interacts selectively with central AMPA/kainate glutamate receptors |
|  | (-)-Epinephrine bitartrate | Adrenaline bitartrate |  | Agonist |  | Endogenous hormone and neurotransmitter |
|  | 2-Chloroadenosine triphosphate tetrasodium | 2-Chloro-ATP tetrasodium |  | Agonist | P2Y | P2Y receptor agonist |
|  | (-)-alpha-Methylnorepinephrine | Levonordefrin; (-)-3,4-Dihydroxynorephedrine |  | Agonist |  | Active enantiomer; adrenoceptor agonist; vasoconstrictor; antihypertensive |
|  | Muscimol hydrobromide | 3-Hydroxy-5-aminomethylisoxazole hydrobromide |  | Agonist | GABA-A, GABA-C | GABA-A receptor agonist, water soluble |
|  | Histamine dihydrochloride | 1H-Imidazole-4-ethanamine dihydrochloride |  | Agonist |  | Endogenous neurotransmitter |
|  | (+)-cis-Dioxolane iodide | L(+)-cis-2-Methyl-4-trimethylammoniummethyl-1,3-dioxolane iodide |  | Agonist | Muscarinic | High affinity muscarinic acetylcholine receptor agonist |
|  | (±)-Epinephrine hydrochloride | (±)-Adrenalin hydrochloride |  | Agonist |  | Adrenoceptor agonist |
|  | L-Glutamic acid hydrochloride | S(+)-1-Aminopropane-1,3-dicarboxylic acid hydrochloride |  | Agonist |  | Endogenous excitatory amino acid neurotransmitter |
|  | OXA-22 iodide | cis-2-Methyl-5-trimethylammoniummethyl-1,3-oxathiolane iodide |  | Agonist | Muscarinic | Potent muscarinic acetylcholine receptor agonist |
|  | N-Methyldopamine hydrochloride | Epinine hydrochloride; Deoxyepinephrine hydrochloride |  | Agonist |  | Dopamine receptor agonist |
|  | FPL 64176 | 2,5-Dimethyl-4-[2-(phenylmethyl)benzoyl]-1H-pyrrole-3-carboxylic acid methyl ester |  | Activator | L-type | Potent L-type Ca^2+^ channel activator |
|  | P1,P4-Di(adenosine-5')tetraphosphate triammonium | Ap4A |  | Inhibitor |  | Dinucleoside polyphosphate that competitively inhibits ADP-induced platelet aggregation |
|  | Kainic acid | 2-Carboxy-3-carboxymethyl-4-isopropenylpyrrolidine |  | Agonist | Kainate | Excitatory amino acid receptor agonist selective for the kainate glutamate receptor subtype; conformationally restricted analog of L-glutamic acid |
|  | 2-Methylthioadenosine diphosphate trisodium | 2-(Methylthio)-adenosine 5'-trihydrogen diphosphate trisodium |  | Agonist | P2Y | P2Y receptor agonist |
|  | Phenylephrine hydrochloride |  |  | Agonist | alpha1 | alpha1 Adrenoceptor agonist; mydriatic; decongestant |
|  | Loxapine succinate |  |  | Antagonist |  | Dibenzoxazepine antipsychotic agent |
|  | Oxotremorine methiodide | N,N,N-Trimethyl-4-(2-oxo-1-pyrrolidinyl)-2-butyn-1-ammonium iodide |  | Agonist | Muscarinic | Nonselective muscarinic acetylcholine receptor agonist |
|  | Spiperone hydrochloride | R 5147 hydrochloride; Spiroperidol hydrochloride |  | Antagonist | D2 | Selective D2 dopamine receptor antagonist. |
|  | Sanguinarine chloride | 13-Methyl-[1,3]benzodioxolo[5,6-c]-1,3-dioxolo[4,5-i] phenanthridinium chloride |  | Inhibitor | Na^+^/K^+^ ATPase | Inhibitor of Mg^2+^ and Na^+^/K^+^-ATPase; isolated from the leaves and stems of Macleaya cordata and microcarpa |
|  | (+)-Quisqualic acid | L(+)-alpha-Amino-3,5-dioxo-1,2,4-oxadiazolidine-2-propanoic acid |  | Agonist | AMPA | Active enantiomer of quisqualic acid;  excitatory amino acid at glutamate receptors; anthelmentic agent |
